# Removal of radionuclides from acidic solution by activated carbon impregnated with methyl- and carboxy-benzotriazoles

**DOI:** 10.1038/s41598-020-68645-4

**Published:** 2020-07-16

**Authors:** Muna A. Abu-Dalo, Svetlana Nevostrueva, Mark Hernandez

**Affiliations:** 1 Chemistry Department, Jordan University of Science and Technology, P.O Box 3030, Irbid, 22110 Jordan; 2 Department of Civil and Environmental Engineering, University of Colorado at Boulder, Campus Box 428, Boulder, CO 80309-0428 USA

**Keywords:** Environmental chemistry, Pollution remediation, Environmental sciences

## Abstract

The removal efficiencies of metals commonly used to model the fate and transport of aqueous uranium and radioactive its daughter products, were observed on activated carbons impregnated with different benzotriazole derivatives. Acidic solutions containing U(VI), Sr(II), Eu(III), and Ce(III) were used to determine the immobilization potential of carboxybenzotriazole (CBT) and methylbenzotriazole (MeBT), where these derivatives were sorbed to different types of granular activated carbon (GAC). This sorption behavior can be predicted by Redlich–Peterson model. Flow-through column tests showed that the immobilization of uranium and some of its daughter products, significantly improves in response to oxidized GACs saturated with carboxybenzotrzole (CBT), which reached a maximum elimination for U(VI) at 260 BV, Eu(III) at 114 BV, Ce(III) at 126 BV, and Sr(II) at 100. MeBT significantly desorbed from GAC under acidic conditions. Trace amounts of CBT were observed in some column effluents, but this did not appear to alter the effectiveness of metal removal, regardless of the model radionuclide studied. These results suggest that enhanced immobilization of selected metals on GAC, can be achieved by impregnating oxidized activated carbon with carboxylated benzotriazoles, and that metal removal efficiency on this media, is related to their valence and ionic radius in acidic environments.

## Introduction

Significant sources of aqueous radioactive metals are associated with nuclear reactor operations, industrial radioisotope research, and health care applications^1^. The treatment of wastewaters containing uranium and its radioactive daughter products continues to receive significant attention. Hooper and Kavanaugh^2^ recognize the dominant regulatory principles governing radionuclide treatment that predominate today: as low as reasonably practicable (ALARP) and best available technology not entailing excessive cost (BATNEEC). A new generation of technology is needed for more effective immobilization of heavy radionuclides.

The methods used to treat metal bearing radioactive wastewaters include chemical precipitation^3^, solvent extraction^4^, extraction chromatography (EXC)^5,6^ ion exchange^7^, membranes^8,9^, and sorption processes^1,10–13^. Because of costs and reliability, hydroxide precipitation remains one of the most common techniques for radioactive metal removal. However, where multiple metals are present, the efficiency of hydroxide addition can be compromised by solubility product and solute competition; thus, precipitating mixed metal wastes has significant treatment challenges, including the generation of low-density sludge^14^. Sulfide precipitation is practiced in this sector, and has the following advantages over hydroxide precipitation: (i) better performance over broader pH ranges; and, (ii) better sludge dewatering characteristics^15^. However, there are significant operational risks associated with sulfide precipitation, including H_2_S gas generation and colloid formation that poses sedimentation and membrane fouling challenges^16^.

Chelating agents, including trimercarptotriazine, thiocarbonates and dimethyldithiocarbamates have been employed to enhance metal radionuclide separation performance. In this context, Cheng et al.^17^ prepared “chelating sponges” by grafting polyvinyl alcohol on different supports, but the performance of these sponges was particularly sensitive to depressing pH levels^18^. Competing solubility of metal hydroxides and interferences from ubiquitous complexing agents (e.g. (EDTA)) remains a challenge for precipitation approaches.

Ion exchange is commonly employed to decontaminate water containing radionuclides, but this approach also has limitations since heavy metal uptake and carrier stability are also sensitive to pH, temperature and contact time in ranges that compromise performance. Various ion exchange resins have the ability to substitute their alkali cations with heavy metals dissolved in industrial wastewaters. Synthetic resins are often preferred because they can be tailored to specific metals and their performance can be predicted under a broad range of water quality conditions^7^. Conventional ion exchange and sorption technologies adapted for aqueous radionuclide immobilization, often employ costly polymeric materials that can be compromised in acidic pH ranges, as was well as by the radiation emitted from the very metals they sequester.

Sorption is recognized as one of the more cost-effective methods for (pre-) treating metal-containing industrial wastewaters. Lower-cost sorbents, including a new generation of acid-stable activated carbon, has revitalized research in this arena. In a comprehensive study conducted by Strelko et al.^19^, three activated carbons were tested for their removal of radioactive^137^Cs, ^239^Pu, ^241^Am, and ^90^Sr. The following investigations also observed enhanced sorption of different radioactive isotopes on specially prepared GACs: ^137^Cs and ^90^Sr^20^, Th(IV)^21^, uranium (VI)^22^ and, ^(152+154)^Eu and ^65^Zn on acid-oxidized GAC^23^. Several studies concur that stable Eu (III) isotopes can serve as an analogue for the treatment of radioactive Am and Ac, while stable Ce(III) can serve as an analogue for the behavior of Pu (III)^24,25^.

Naturally occurring zeolites have been extensively studied for their potential to remove radionuclides from aqueous solutions. A study by Fang et al.^7^, found that a natural zeolite (4A) has a good ability to treat wastewaters containing radioactive isotopes of Sr^2+^, Cs^+^ and Co^2+^, with sorption accounting for 90% removal in near-neutral pH ranges. Chitosan-based sorbents have attracted increasing attention in water and wastewater treatment due to its structure and physicochemical properties, as well as their abundance and low cost. While their sorption capacity of these materials is relatively low, it can be improved by functionalization with strong complexing agents^26,27^. In recent study, magnetic amidoxime functionalized chitosan beads, were developed for uranium removal^28^. The adsorption capacity of uranium was observed to be 117.65 mg/g at pH 6. Romanchuk et al.^29^ used graphene oxides (GO) to remove radionuclides from acidic wastewaters (pH < 2); this study described heavy actinide sorption kinetics on GO surfaces, suggesting that GO is more effective for the removal of transuranic elements from wastewater than many conventional sorbents. However, GO has the disadvantage of relatively low adsorption efficiency, although GO performance has been reportedly enhanced by chemical functionalization^30–32^.

Therefore, there is still a need to explore innovative adsorbents with improved sorption capability and relatively low cost. Some researchers have demonstrated that impregnating GACs with organic compounds that coordinate heavy metals, can improve their ability to sequester metals from acidic solution. These organic compounds include the following coordinating agents, which were pre-sorbed to activated carbon prior to their exposure to metal-containing wastewater: tartrazine^33^; ethylene-diaminetetracetic acid (EDTA)^34^; benzotriazoles^35^; chitosan^36^; pyridines^37^; 8-hydroxy quinoline-5-sulphonic acid^38^; alginate^39^; tannic acid^40^, and different ionic surfactants^41^. Magnesium has been used in this scenario as well^42^. The salient theme circumscribing this approach follows: metal-coordinating agents must have a moiety that sorbs to activated carbon, while concomitantly retaining the ability to form strong complexes with metal ions.

In a series of lab-scale experiments, AbuDalo et al.^35^ showed that corrosion inhibitors could be applied in a GAC immobilization scenario, where benzotriazoles sorb to activated carbon in a predictable complexing action that sequestered several heavy metals (Cu, Pb, and Zn) from acidic solutions. This was the first study to demonstrate that benzotriazole derivatives can retain their ability to bind metal ions, while simultaneously maintaining a strong sorptive affinity for GAC through a broad pH range. The purpose of this research is to optimize benzotriazole-GAC treatment scenario for the express purpose of sequestering transuranic elements from industrial wastewaters using stable isotopes that are accepted as models for radioactive metals U(VI), Eu(III), Sr(II) and Ce(III). We report here, the engineering and application of an enhanced sorption behavior enabled by a novel combination of activated carbon impregnated with corrosion inhibitors, which is remarkably stable at low pH levels. The granular product, enriched in metal radionuclides, can be easily dewatered and incorporated into cementitious materials or other solids for long-term sequestration from the environment**.**

## Materials and methods

### Benzotriazole derivatives

High purity 5-methylbenzotriazole (MeBT, pK_a1_ = 2.2), and a racemic mixture of 4,5-carboxybenzotriazole (CBT, pK_a1_ = 1.5) isomers were used in this study. Methylbenzotriazole solutions were prepared by dissolving desiccated 5-MeBT powder in ultra-pure water, while shaking for 24 h (pH 5). Carboxybenzotriazole solutions were prepared by dissolving desiccated 4-,5-CBT powder in a solution maintained at pH 12.0 with the addition of NaOH; once dissolved, 1 M HNO_3_ was subsequently added until the pH reached 5.0. These solutions were used to saturate the GACs presented below, prior to their exposure to metal containing wastewaters.

### GAC source and characteristics

Granular MRX-P was obtained from the Calgon Carbon Corporation (Catlettsburg KY, USA) with the following specifications: iodine number 950 mg/g (ASTM D4607), ash 10% (TM-5), moisture as packed 5% (ASTM D2867), abrasion resistance 70% (TM-9), butane adsorption 210 mg/g carbon (ASTM D5228), fraction of 10 × 30 with minimum 40% and maximum 59% passing US sieve series (TM-8).

### GAC modification

GAC was modified as follows: oxidation with 20% HNO_3_ (Fisher Scientific, USA) at 90 °C for 8 h using the method previously described by AbuDalo et al.^35^. pH was measured using Orion meter model 525A, confirming that pH 3.5 was achieved by repetitive washing of oxidized GACs with ultrapure water (Milli-Q water; Billerica MA, USA). Subsequently, the oxidized versions of Calgon MRX-P (referred to herein as MRX-Pox) was dried at 90  °C for 2 days, and kept in a desiccator until used.

### GAC characterization

#### GAC pore structure

The specific surface area and pore volumes of the MRX-P and MRX-Pox as used here, was determined by standard gas adsorption methods. Using an adsorption model developed by Brunauer, Emmett and Teller (BET)^43^, the surface area of the GAC was estimated; these isotherm data were employed to assess the total pore volume of the GAC, using a conversion factor for gaseous to liquid nitrogen. These measurements were performed at the Material Synergy Laboratory in Ventura CA, USA, using a Qsurf M3 analyzer and a Micrometrics (N_2_) analyzer.

#### Elemental analysis

Carbon, hydrogen, nitrogen and sulfur contents in respective activated carbons were determined using a 2400 Perkin–Elmer CHNS Analyzer at Elemental Analysis, Inc. (Lexington KY, USA). This analyzer employs combustion to convert the sample elements to simple gases, (CO_2_, H_2_O, N_2_ and SO_3_), which were separated under steady state conditions, and measured by thermal conductivity. Ash content of the GACs was gravimetrically determined at Elemental Analysis, Inc. (Lexington KY, USA). A desiccated 10 mg sample of each GAC was weighed in preconditioned platinum boats, where GAC was covered with sulfuric acid and heated over > 700 °C until a constant weight was achieved.

#### Scanning electron microscopy

The low voltage scanning electron microscope in the Nanomaterials Characterization Facility (NCF) Laboratory at the University of Colorado, was used to image GAC grains. The low vacuum mode (LV) of this thermal emission microscope (JSM-6480LV) allowed observation of GAC samples with minimal surface charging. A secondary electron detector with a backscatter electron detector was used at voltages between 0.3 to 30 kV.

#### Measurement of point zero charge

The point of zero charge for the respective GACs were determined by the indirect mass titration (drift) method, included sodium chloride as the governing electrolyte as modified by Khan and Wahab^44^. Each GAC cohort was thoroughly washed with ultra-pure water, followed by a dilute sodium hydroxide rinse (pH 10) to neutralize any residual surface-associated acid; these particles were subsequently soaked in HCl for 24 h. The pH of test solutions was adjusted in 0.005 M NaCl through a pH range between 1.9 and 10.9 as adjusted by 0.5 M HCl or 0.5 M NaOH, where (0.06 g) of GAC was added into 20 mL of the pH-adjusted solutions in ultra-clean, virgin glass vials, subsequently equilibrated for 24 h. The final pH was measured and plotted against the initial pH of the respective preparation; the pH at which the plotted values cross the initial pH is defined as the pH of zero-point charge (pH_pzc_).

### HPLC and ICP/AES

A Spectra-Physics high performance liquid chromatograph (HPLC, model SP8800) fitted with a UV detector (λ = 254 nm) was used for analysis of 5-MeBT and 4,5-CBT isomers, which were separated on a Zorbax Rx-C8 4.6 × 250 mm column from MacMod Analytical, Inc. (Chadds Ford PA, USA). The eluent consisted of a phosphate buffer mixed in a 70:30 ratio with HPLC grade acetonitrile at a flow rate of 1.0 mL/minute with a sample injection volume of 200 μL. Retention time of 4-CBT and 5-CBT was 3.8 min and 4.1 min, respectively, and 8.1 min for 5-MeBT. Detection limit for all benzotriazole derivatives was below 0.1 ppm. All samples were centrifuged at 10,000×*g* for 5 min prior to analysis.

Metal analysis was performed using inductive coupled plasma/atomic emission spectroscopy a USEPA certified laboratory in the Geology department at University of Colorado (Boulder, Colorado, USA). Chemicals for HPLC analysis, metals, acids/bases and ICP/AES standards were obtained from Fisher Scientific (Pittsburgh PA, USA) and Sigma-Aldrich (St. Louis, MO, USA).

### Method for determining behavior of benzotriazoles on oxidized and non-oxidized carbons

Batch studies were executed to identify the MeBT and CBT loadings near the sorptive capacity of MRX-P, and MRX-Pox, as judged by Redlich-Peterson isotherm modeling^45^, following the procedure described Ababneh et al.^46^. Based on solubility, MeBT concentrations near 5 g/L, and CBT concentrations near 10 g/L were isolated in three independent replicated microcosoms (3 ×), each containing between 0.6 g to 2.5 g of five different GAC preparations; 75-mL aliquots of MeBT/CBT solution, containing pre-determined GAC masses were placed in 250 mL Erlenmeyer flasks, served as the microcosms. Once the benzotriazole solutions were introduced, they were sheilded from light and allowed to equilibrate on a shaker table at 25 °C. The equilibrium time was determined in prior experiment by simple time-series analysis of soluble benzotriazole derivative, the minimum time required for complete sorption was determined. Each experiment reached equilibrium before 4 days, using a criteria of no more than 4% change in aqueous concentration during the observation period. Following equilibration, the samples were centrifuged and the supernatant was analyzed for 5-MeBT, 4,5-CBT by HPLC method.

Similar batch experiments were conducted for testing thorium sorption on activated carbons impregnated with MeBT/CBT. This metal was chosen as an example to study the complexation behavior and adsorptive interactions when benzotriazole isomers were bound to different radionuclides. During the process, the carbons were allowed to equilibrate with benzotriazoles while shaking for two days with subsequent addition of metal-containing solution. The resulting two-phase metal impregnation systems (2PMI) mixture was placed on a shaker table at 25 °C for 2 days. Thorium concentrations were varied between 10 and 100 mg/L, MeBT/CBT were presented in the amount of 0.3 g/L and mass of carbon was 1.5 g/L, which resulted in different metal-benzotriazole ratios. Control systems consisted of otherwise identical systems that did not include MeBT/CBT on GAC. Following equilibration, the samples were centrifuged and the supernatant was analyzed for 5-MeBT, 4,5-CBT by HPLC method and thorium concentration was assessed via ICP/AES in CU Geology Department.

In previous investigation, Redlich-Peterson isotherms was best-described benzotriazoles partitioning on MRX-P^46^ and could be successfully utilized to model sorption of Th (IV) on oxidized (MRX-Pox) and non-oxidized (MRX-P) carbon in the presence and absence of sorbed benzotriazole derivatives. It can be described as follows:

1 $$q_{eq} = \frac{{K_{R} *C_{eq} }}{{1 + \alpha_{R} C_{eq}^{\beta } }}$$

q_eq_(mg/g)—liquid phase concentrations of adsorbate at equilibrium, C_eq_ (mg/L)—solid phase concentrations of adsorbate at equilibrium, K_R_ α_R_, and β (β $\le$ 1) are Redlich–Peterson isotherm constants.

### Flow-through column experiment design

One gram of desiccated MRX-P or MRX-Pox was packed into a Teflon tube with an internal diameter of 4.9 mm with a 120 mm carbon bed length; both ends were packed with a thin layer of glass wool; pore volume was approximately 2.2 mL. The following describes the six types of columns used for observing metal removal capacity: (1) columns packed MRX-P; (2) columns packed with MRX-Pox; (3) columns packed with MRX-P, saturated with MeBT (MRX-P + MeBT); (4) columns packed with MRX-Pox, saturated with MeBT (MRX-Pox + MeBT); (5) columns packed with MRX-P, saturated with CBT (MRX-P + CBT); and, (6) columns packed with MRX-Pox, saturated with CBT (MRX-Pox + CBT). During these experiments, pH was controlled between 1.0 and 4.5.

Selected GAC columns were loaded as follows: for MeBT, 125 mL of a 5 g/L MeBT solution at pH 5.0, was introduced at a flow rate of 10 mL/h using a peristaltic pump. The same loading technique was applied to saturate selected columns with 125 mL of 10 g/L CBT solution at pH 5.0. Once loaded, columns were dried at 90 °C for 2 days and the glass wool replaced to avoid any potential benzotriazole residuals on the containing materials. Sorption capacity of the different GACs was estimated by comparing composite measurements of the influent and effluent benzotriazole concentrations.

Acidic metal-containing solutions (0.5 mmol) were continually introduced to the respective columns in an upflow mode at 0.3 mL/min flow rate (empty bed contact time of 8.3 min). The solutions were buffered with 5 mmol of either chloroacetic or maleic acid, and adjusted to challenge pH by 1 M NaOH or 1 M HNO_3_. The effluents were collected in 7 mL lots using a radial fraction collector fitted with a 136 virgin glass tubes (Eldex Laboratories Inc.).

According to the method introduced by Omar and Moloukhia^23^, the retention capacities under the different pH conditions tested are calculated as follow:

2 $$break\,through\, capacity={V}_{50\%}\frac{{C}_{0}}{m}$$

where V_50%_ (mL)—volume passed at 50% total capacity, C_0_ (mg Metal/L)—initial metal concentration, m (g GAC)—bed material mass.

## Results and discussion

### Effect of oxidation on carbon microstructure

**Figure. 1:**
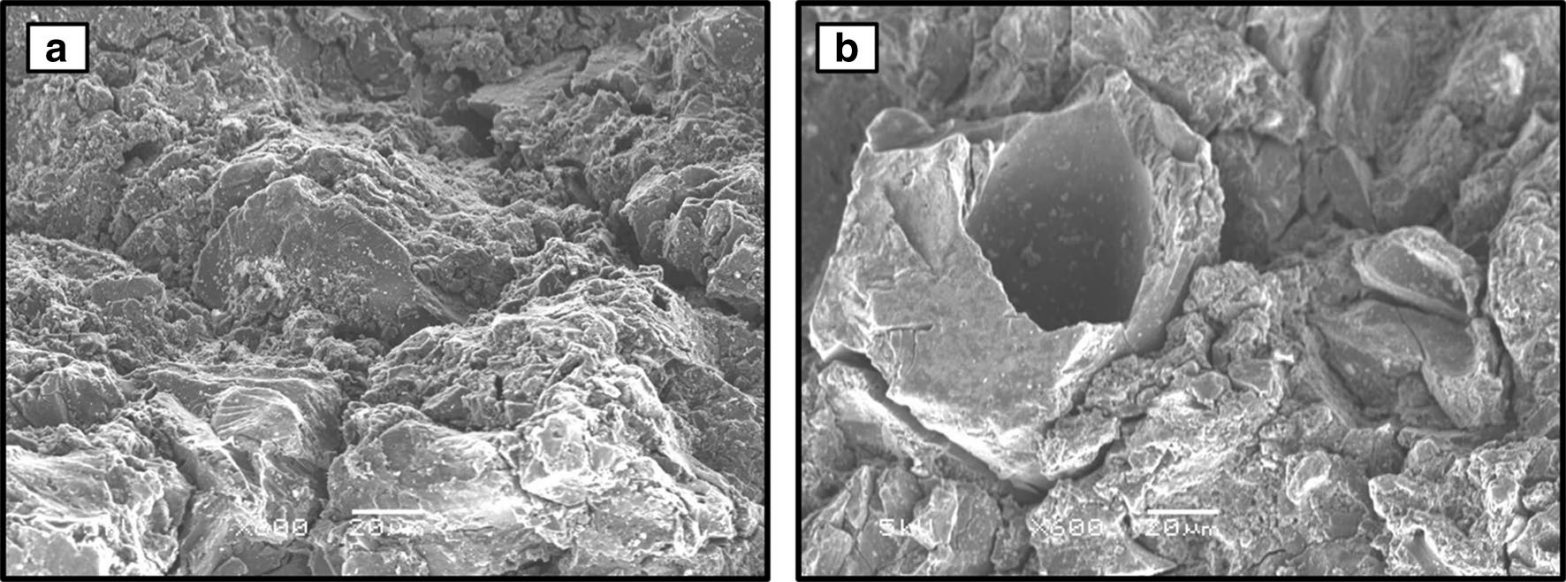
Electron microscope photographs showing typical surface and pore structure of GAC. (**a**) MRX-P (magnification × 600) and (**b**) MRX-Pox (magnification × 600).

When oxidized with acid, MRX-P carbon realized a significant reduction in specific surface area—yet the oxidation process did not appear to change the carbon pore structure. Representative surfaces of MRX-P and MRX-Pox grains were observed in accordance with Qiao et al.^47^, who reported the use of scanning electron microscopy (SEM) for associating surface modifications with sorption behavior. SEM images show distinct difference in surface roughness and porosity in response to acidification, yet there was no evidence for the surface precipitation of metal on any GAC observed by SEM. Representative SEM images are presented in Fig. 1; similar observations were documented by Goel et al.^48^ for acid treated GACs derived from coconut. Granular activated carbon can exhibit acidic character when modified by a strong oxidant such as nitric acid^49–51^; these GACs are often referred to as “*L-type*”^52^. Their acidic nature has been primarily attributed to oxidized surface associated functional groups^﻿53﻿^.

Oxidized MRX-P carbon realized a significant reduction in specific surface area from 985.5 m^2^/g to 851.6 m^2^/g. Additionally, there was a decrease observed of total pore volume from 0.588 cm^3^/g for MRX-P to 0.522 cm^3^/g for MRX-Pox, respectively. Analogous effects were observed by Goel et al.^48^ and Strelko et al.^51^, where commercial GACs were acid treatments induced significant surface area and pH_PZC_ changes. Elemental analysis performed on MRX-P and MRX-Pox is summarized in Table 1, which is consistent with significant surface oxidation^35^.

**Table 1:** Summary of elemental analysis for selected carbons.

GAC sample	Oxygen (wt%)	Carbon (wt%)	Hydrogen (wt%)	Nitrogen (wt%)	Sulfur (wt%)	Ash (wt%)
MRX-P	1.34	83.89	0.71	0.42	0.30	6.80
MRX-Pox	12.97	65.43	1.57	0.74	0.30	4.84

**Figure 2:**
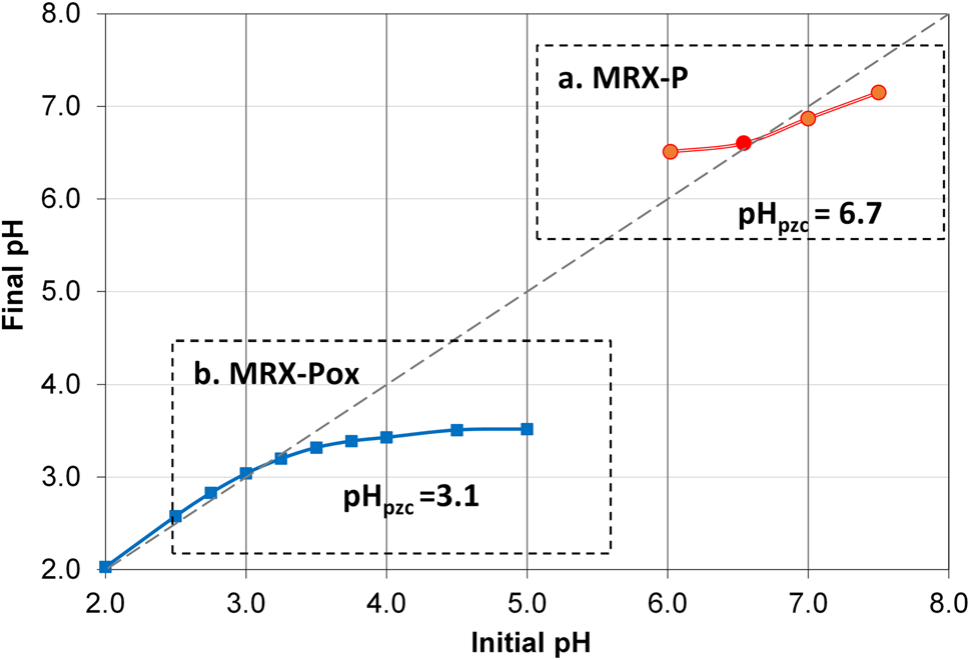
Determining pH of point of zero charge of two candidate GACs by the pH drift method: (**a**) MRX-P; and, (**b**) MRX-Pox.

The pH at zero-point charge (pH_pzc_) is a master variable influencing sorption processes; this affects the interaction potential between the sorbent and sorbates. The pH_pzc_ of the GAC was observed to drop from 6.7 to 3.1 in response to acid oxidation (Fig. 2). Collectively, these results suggest GAC surfaces are positively charged at pH < pH_pzc_ and negatively charged above the pH_pzc_ level. Electrostatic attraction (or repulsion) of dissolved metal species with GAC is sensitive to this parameter^54^; thus, carbon with lower pH_pzc_ values should exhibit higher sorption capacities for retaining metals in response to depressing pH levels.

### Sorption of MeBT/CBT on oxidized and non-oxidized carbons

**Figure 3:**
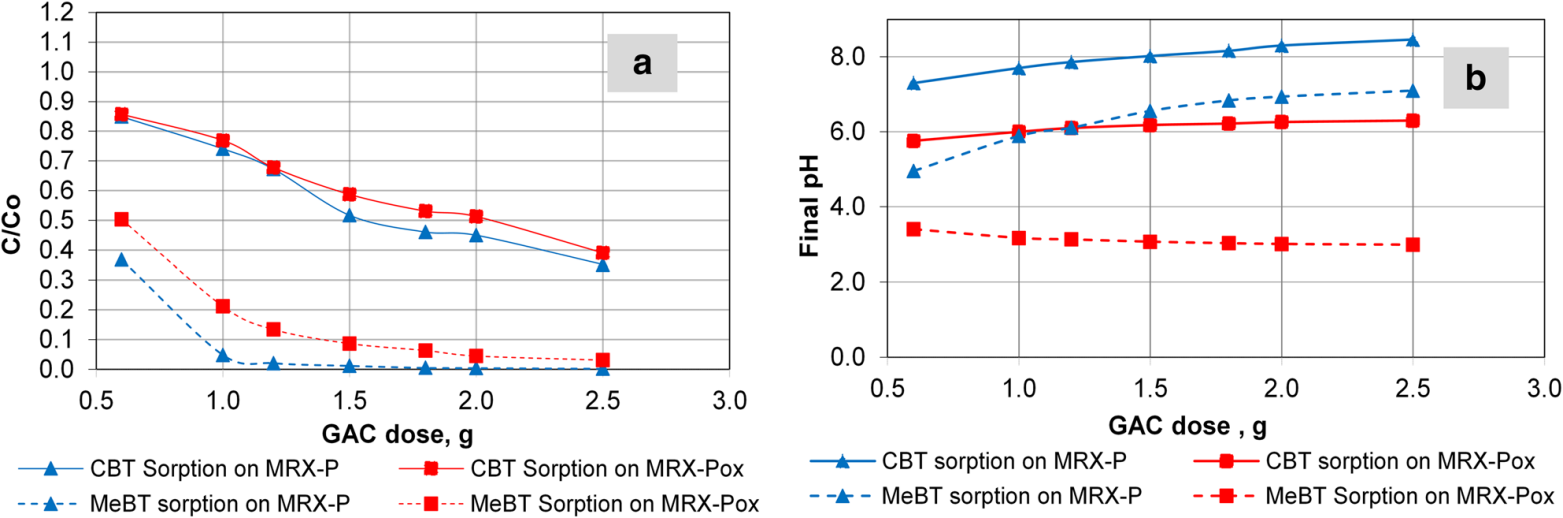
(**a, left**) Fraction of amount adsorbed of 5-MeBT and 4,5-CBT (C/Co); (**b, right)** pH changes in response to MeBT and CBT sorption. Initial concentration of MeBT 5 g/L, CBT 10 g/L; GAC mass ranges between 0.6 g and 2.5 g.

The sorption behavior of benzotriazole derivatives on the different GACs tested here are summarized in Fig. 3. The pH_pzc_ for MRX-P was 6.7, which is significantly above the pH of the solution used to load the benzotriazole derivatives on the activated carbons (pH 5.0). The pH_pzc_ for MRX-Pox was 3.1, which is below the loading solution pH. MRX-P showed better affinity for MeBT than CBT, which did not saturate the GAC. The relatively smaller size of the MeBT molecule, as well its pK_a_, was likely associated with the higher MeBT sorption capacity observed as compared to CBT. The differences between CBT’s sorption potential on oxidized and unadulterated MRX-P are likely associated with electrostatic attractions between the deprotonated carboxyl groups and a preponderance of positively charged sorption sites under the column “loading” conditions. A parallel result was reported by Li et al.^55^ when comparing the sorption behavior of trichloroethene (TCE) and methyl tertiary-butyl ether (MTBE) on 15 different GAC types. Their studies demonstrated that activated carbon fibers (type ASF), with similar pore structures but different surface chemistries, exhibited similar TCE sorption capacities. These authors suggested that TCE cannot form H-bonds and primarily interacts with basal activated carbon planes via van der Waals forces. On the other hand, MTBE, as hydrogen-bond acceptor, had a relatively higher affinity for activated carbons that were more enriched with oxygen-containing functional groups. Moreover, Knappe^56^ summarized the physico-chemical properties of 110 organic pollutants for their sorption potential on different GACs based on their molecular weight, molar volumes and solubilities.

Analogous outcomes from these experiments are consistent with the pH-mediated sorption behavior observed here (Fig. 3b). MeBT and CBT sorption response, and the equilibrium pH of oxidized carbon (MRX-Pox), remained acidic, which suggested this GAC is representative of L-type carbon. Sorption of MeBT, compared to its initial pH (5.0), resulted in equilibrium pH near pH 3.1 (MRX-Pox carbon). In the case of CBT, equilibrium pH did not change substantially, when compared to the initial pH of 5.0. An analogous trend was observed in the benzotriazole sorption experiments performed on unmodified MRX-P, which was chosen to contrast H-type GAC behavior (MRX-P, more basic carbon). In these batch tests, the acid modified MRX-Pox proved to have a superior sorption capacity for benzotriazoles in acidic media; thus, MRX-Pox was chosen for more extensive metal immobilization potential observations described below.

### Thorium (IV) sorption on modified GACs impregnated with benzotriazole derivatives

**Figure 4:**
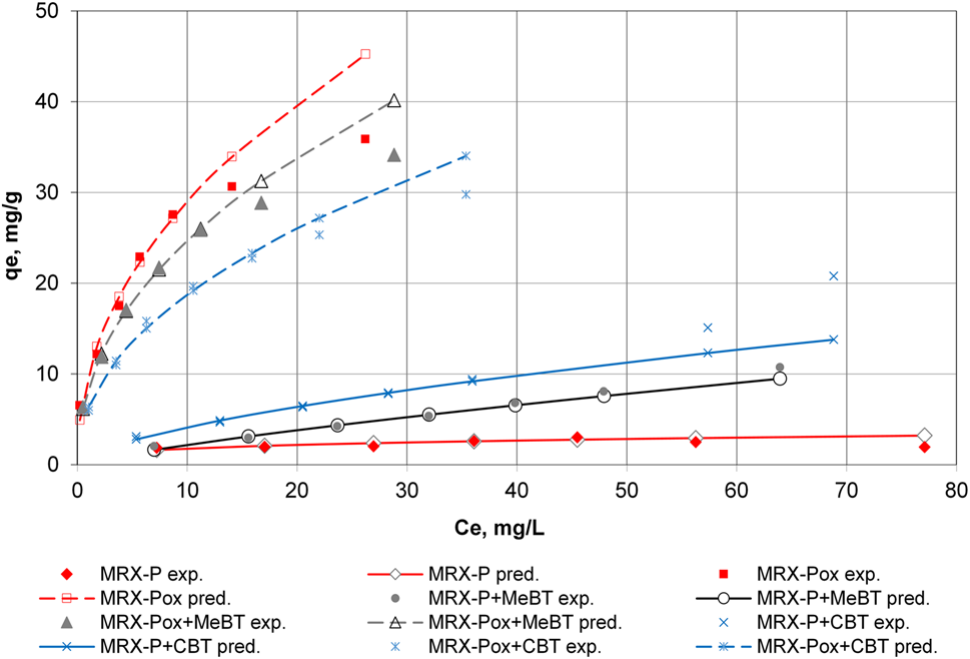
Comparison of observed and modeled sorption of Th(IV) on GAC impregnated with benzotriazoles in acidic aqueous solution. Initial thorium concentrations were varied between 10 and 100 mg/L, initial pH 2.8, on 1.5 g of oxidized and non-oxidized MRX-P carbons in the presence or absence of MeBT and CBT.

Thorium was chosen as an example to study the complexation behavior and adsorptive interactions of this metallic radioactive element, when benzotriazole isomers are coordinating different radionuclides. The sorption equilibrium of Th(IV) on GAC impregnated with different benzotriazole derivatives is presented in Fig. 4.

Oxidized activated carbon (as a representative of L-type carbon group) demonstrated better sorption capacity for Th(IV) than its non-oxidized counterpart (H-Type). This is likely due to changing surface chemistry of GACs through basic oxidation^57^. Similar results were obtained by Selomulya et al.^58^ for Cr(VI) sorption performed on wood (L-type) and coconut shell (H-type) activated carbons. Stronger oxidation agents such as nitric acid were applied in later experiments in order to introduce more surface acidic groups.

Considering this trend, it was also observed that MRX-Pox + CBT was able to remove a smaller amount of Th(IV) ion from solution than methylated benzotriazole (MeBT). This is probably because MeBT on the surface of this carbon is held via hydrophobic interactions with its methyl group than the more polar carboxylated CBT. Moreover, the complexation constants for MeBT and Th(IV) may be significantly different than CBT-Th(IV) values in this acidic solution. The opposite performance was observed for the behavior of unadulterated MRX-P carbon, regardless of loading with MeBT and CBT. This can be explained by the presence of diverse functional groups on the GAC surface, which has different dissociation constants and pH_pzc_ value of 6.7 (versus 3.1 for oxidized carbon).

Redlich-Peterson modeling^45^ was in good agreement with both oxidized and non-oxidized forms of MRX-P carbon, when pre-conditioned with benzotriazoles, could sequester Th(IV) from acidic solutions (Fig. 4). This modeling approach may be useful for describing the affinity of these GACs for heterocyclic ligands and the ability of these immobilized ligands to subsequently enhance GAC’s capacity to remove selected metals in certain industrial applications, notably including acidic conditions where metals are ionized and mobile. The constants describing Redlich–Peterson isotherms where GAC is pre-conditioned with triazoles are summarized in Table 2.

**Table 2:** Redlich–Peterson constants for Th(IV) 2PMI challenges on 6 GACs.

Carbon	K_R_	a_R_	β	R^2^
MRX-P	1,161.4	1,249.0	0.72	0.750
MRX-Pox	2,261.4	226.1	0.54	0.982
MRX-P + MeBT	1.1	2.2	0.25	0.982
MRX-Pox + MeBT	1,294.6	151.2	0.54	0.999
MRX-P + CBT	320.5	325.1	0.38	0.992
MRX-Pox + CBT	1,288.5	203.8	0.53	0.992

Among the relatively limited body of work on the removal of thorium from contaminated environments, confirmed thorium immobilization on acid-modified GAC surfaces, performed better at pH 2.8 when compared to unmodified GAC and zeolites^21^. The adsorption isotherms of copper observed by Doss and Natarajan^59^ on F400 GAC pre-adsorbed with oxine and 2-methyloxine behaved similarly. They found that the quantity of copper near the saturation level of these ligands was not in otherwise typical stoichiometric ratios 1:2 (metal:ligand) since a large portion of oxine and 2-methyloxine adsorbed on the GAC was in the micro pores where sufficient molecular orientation of the ligands was not available complex with the dissolved metal ions. Gabaldon et al.^60^ reported that in most cases, batch adsorption isotherms underestimate the metal retention observed in otherwise identical column experiments. Discrepancies between these formats were explained on the basis of combined removal mechanism including adsorption and surface precipitation processes. According to Reed et al.^61^, possible reasons for GAC surface precipitation of the ligands, and/or ligand–metal complexes include but are not limited to the following factors: surface pH is higher than solution pH; the surface acts as a nucleus for precipitation; and/or OH-accumulates in the carbon pores. These effects may become higher as the adsorbate concentration approaches saturation concentration. In any case, surface precipitation was not observed in this or any other batch adsorption experiments, as judged by electron microscopy.

### Retention of model radionuclides U(VI), Sr(II), Eu(III), and Ce(III) in flow through columns

The performance of benzotriazole impregnated GACs for the following model radionuclides were investigated in column experiments: U(VI), Sr(II), Eu(III), and Ce(III). Six carbon types were investigated in column experiments with the following mass fractions of adsorbed MeBT and CBT relative to 1 g of GAC as follows: MRX-P (control); MRX-Pox (control); MRX-P:0.36 MeBT; MRX-Pox:0.3 g MeBT; MRX-P:0.19 CBT; MRX-Pox:0.17 CBT. Sorption behavior was investigated at pH levels in the range between 1.0 and 4.5, where individual metal concentrations of 0.5 mmol/L, were used as the challenge solutions.

**Figure 5:**
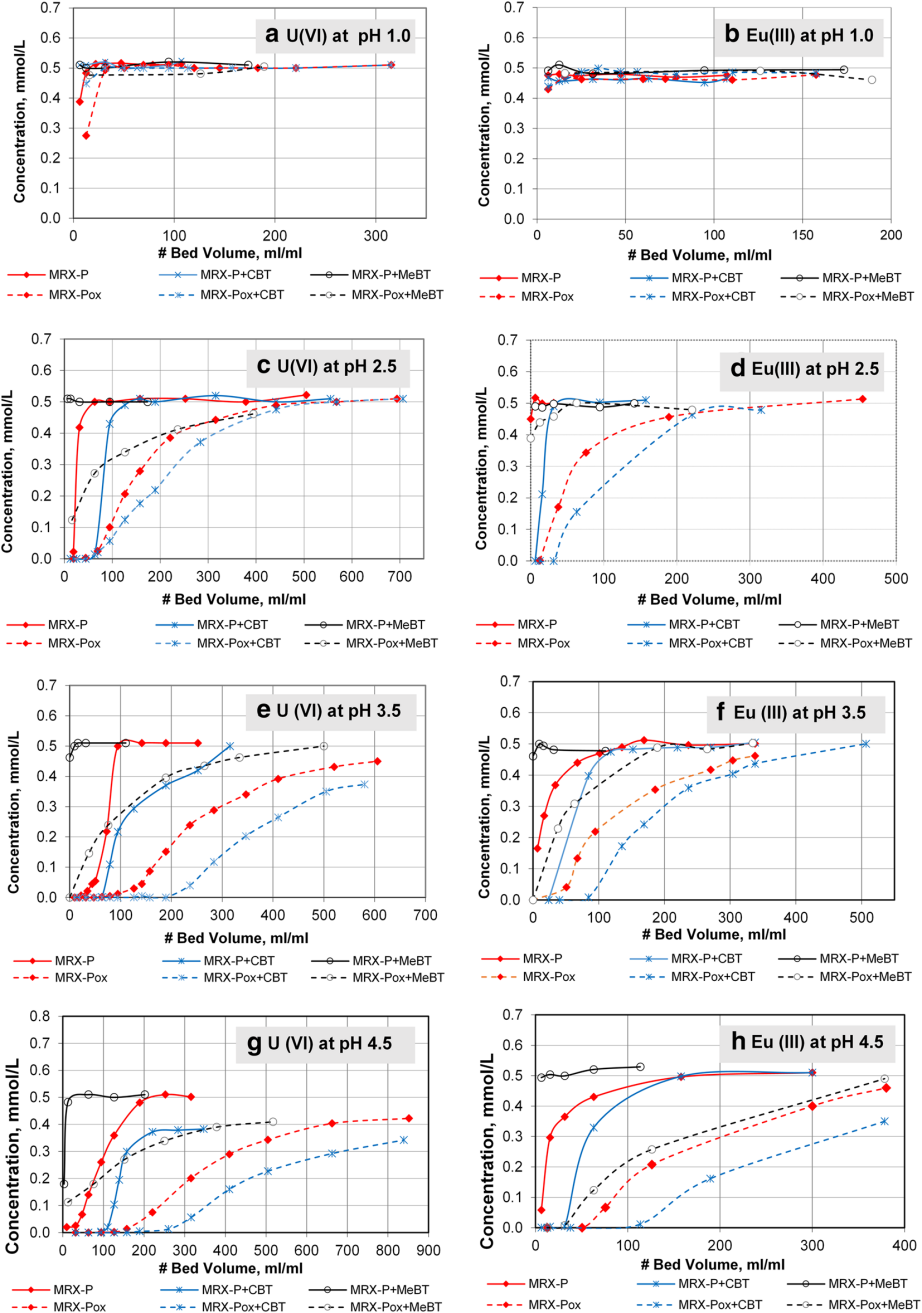
Retention performance of 0.5 mmol/L of: U(VI) and Eu(III) on six GAC types at pH 1.0 to 4.5.

**Figure 6:**
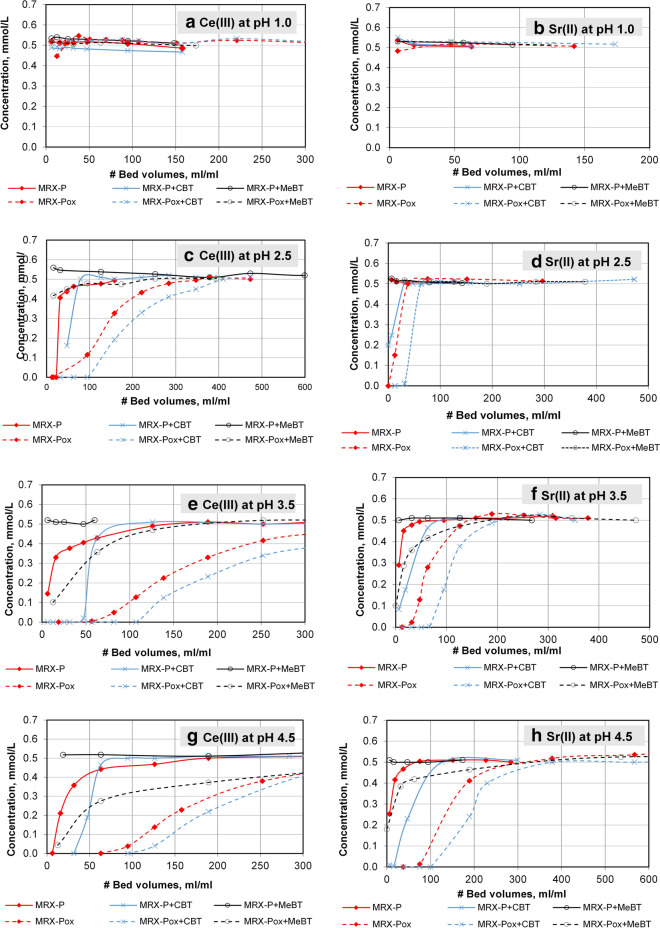
Retention and breakthrough sorption of 0.5 mmol/L of: Ce(VI) and Sr(II) on six GAC types at pH 1.0 to pH 4.5.

At pH 1.0, there was no appreciable metal retention by any of the GACs tested; their effluent concentration remained identical to their influents for U(VI) (Fig. 5a) Eu(III) (Fig. 5b), Ce(III) (Fig. 6a) and Sr(II) (Fig. 6b). This outcome supports the concept that functional groups on the GACs’ surfaces as well as the associated benzotriazoles, are positively charged and will coordinate poorly with aqueous metals cations. Metal removal performance appeared inhibited when pH falls below the GAC pH_PZC_ value; column performance in this pH range is likely because MeBT and CBT carry a positive charge below their pK_a1_ (2.2 and 1.5, respectively).

The flow-through column metal retention tests conducted at pH 2.5 are summarized for U(VI) (Fig. 5c), Eu(III) (Fig. 5d), Ce(III) (Fig. 6c), Sr(II) (Fig. 6d). The addition of 4,5-CBT to oxidized carbon resulted in notably increased efficiency of model radionuclide removal (U(VI) and Eu(III) (70 and 32 bed volumes (BV) when the challenge solution pH was maintained above its pK_a1_ (1.5). CBT molecules in this pH range are predominantly in their neutral form, which apparently aides their sorption to the near-neutral GAC surface while coordinating these metal cations. The same trend was observed for the removal of Ce(III) and Sr(II) (95 and 30 bed volumes (BV), respectively), whereas carbon impregnated with CBT was less effective for immobilizing Sr(II). Variation in the stability between CBT and divalent- and trivalent-ions could be associated with that difference. Moreover, MRX-Pox + CBT allowed 70 bed volumes (BV) of uranium-laden solution to pass before breakthrough occurred. Parallel tests conducted on MRX-P + MeBT were not effective in metal removal at pH 2.5, since near its first pK_a1_ (2.2), MeBT is on the cusp of changing from positive to neutral. No metal removal was observed for the columns packed with MRX-P, MRX-P + MeBT and MRX-P + CBT carbons at this pH level (2.5), where immediate breakthrough of the cations in the effluent was observed**.**

Column retention behavior at pH 3.5 and pH 4.5 for U(VI) (Fig. 5e), Eu(III) (Fig. 5f), Ce(III) (Fig. 6e) and Sr(II) (Fig. 6f) are summarized. Compared to the breakthrough isotherms at pH 2.5, the following increases in the bed volumes (BV) that completely removed the U(VI) and Eu(III), Ce(III) and Sr(II) at pH 3.5 using MRX-Pox + CBT were observed: from 32 to 85 for Eu(III); from 95 to 108 for Ce(III); and, from 30 to 67 for Sr(II); however, the immobilization performance for soluble uranium tripled from 70 to 200 BV. At this pH level (3.5) some metal sorption with MRX-Pox alone still occurred; however, net retention decreased two fold. In the case of MRX-P + MeBT, some removal of U(VI) was observed at pH 3.5, but columns packed with MRX-P alone showed no reduction at pH 3.5. The addition of CBT to MRX-P resulted in a modest metal elimination, which was most evident for uranium (Fig. 5e). These results are in agreement with those observed for U(VI) removal on Merek GAC, which were executed under acidic conditions at pH between 1.0 and 6.0^22^. These outcomes are consistent with suggestions by SenGupta^62^ and coworkers, who anticipated that metal immobilization potential for oxidized carbons loaded with conventional chelating agents (likely not including benzotriazole derivatives) may be higher compared to their non-oxidized counterparts, under otherwise identical conditions.

The ability of six carbon types to remove selected radionuclide models at pH 4.5 is illustrated in Figs. 5e,f and 6e,f. Likely because this pH level was above pH_PZC_ for MRX-Pox (3.1), significant removal of U(VI), Eu(III), Ce(III) and Sr(II) was observed. MRX-Pox + CBT breakthrough patterns followed the same trend observed at pH 3.5, with performance increases extending from 85 to 114 BV for Eu(III); from 108 to 126 for Ce(III); from 67 to 100 for Sr(II); and, from 200 to 260 for U(VI).

The retention capacities under the different pH conditions tested are calculated according to the method introduced by Omar and Moloukhia^23^ and summarized in Table 3 below.

**Table 3:** Breakthrough capacity for the metal challenges in flow-through column tests at different pH levels (mg Metal/g GAC).

pH	Metal	MRX-P	MRX-Pox	MRX-P + CBT	MRX-Pox + CBT	MRX-P + MeBT	MRX-Pox + MeBT
2.5	Sr(II)	0	1.9	0.5	5.3	0	0
3.5		0.5	5.8	2.5	8.8	0.4	0.9
4.5		0.6	14	4.4	16	0	0.8
2.5	Eu(III)	0.6	9.2	2.5	13.3	0	1
3.5		5.7	19.3	8.8	25.3	0.6	5.7
4.5		2.4	26.3	7.8	36.1	0	16.1
2.5	Ce(III)	0	11	4.4	12.1	0	0
3.5		1.8	23.2	7.4	26.4	0	5
4.5		3.1	26.6	6.6	28.3	0	3.5
2.5	Th(IV)	5.7	39.5	8.3	23.6	2.6	25.3
3.5		13.1	32.2	13.5	42.3	3.7	26
2.5	U(VI)	6.9	38.4	18	47.7	0	11.5
3.5		19.8	67.8	24.4	84.6	1	55.8
4.5		24.5	101.5	33	131	1	47.2

Immobilization performance of these metal radionuclide models significantly improved in response to increasing pH values between 2.5 and 4.5. Similar results were obtained by Strelko et al.^51^ for ^241^Am, ^90^Sr, ^239^Pu, ^237^Np, as well as by Awwad et al.^63^ for Sr(II) adsorption on air-oxidized GAC. This outcome is also in agreement with the studies conducted by Omar and Moloukhia^23^ for the sorption of ^152^Eu and ^65^Zn. However, these studied notably did not include the metal chelating agents introduced here.

The distribution of U(VI), Eu(III), Ce(III) and Sr(II) in aqueous solutions was mainly as positively charged species (i.e., UO_2_^2+^, UO_2_OH^+^, Eu^3+^, Ce^3+^, Sr^2+^ species) at pH < 5.0. Therefore, the increased adsorption of U(VI) > Ce(III) > Eu(III), > Sr(II) on the GACS from pH 1.0 to pH 4.5 could be attributed to the strong surface complexation between positive charged radionuclide species and negatively charged surface of the GACs when the challenge solution pH was maintained above its pK_a1_ (1.5). These results are in agreement with those observed for U(VI) and Eu(III) removal on carbonaceous nanofibers, which were executed under acidic conditions at pH between 1.0 and 11.0^64^. This outcome is also in agreement with the studies conducted by Zhao et al.^65^ for the sorption of U(VI) and Sr(II) on graphene oxide.

The relatively large ionic radius of Sr(II) (1.18 Å) can cause rapid saturation of the adsorbent due to steric effects resulting in a lower adsorption on the adsorbent surface compared to Eu(III) (0.95 Å) and Ce(III) (1.07 Å)^66^.

### MeBT/CBT liberation during flow through column challenges

**Figure 7:**
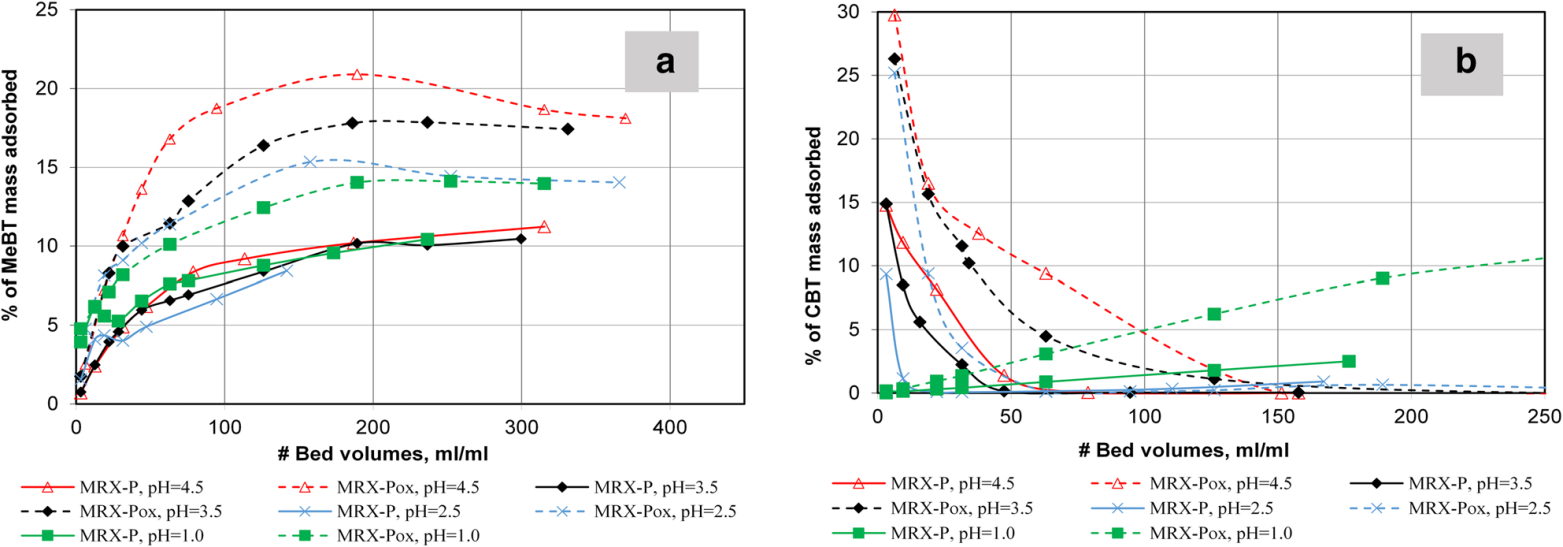
MeBT and CBT liberation from 2PMI columns on oxidized and non-oxidized carbon for Eu(III) challenges at pH range 1.0–4.5.

Figure 7a presents Eu(III) removal performance in the pH range between 1.0 and 4.5. In the case of MRX-P carbon, effluent MeBT increased as more metal-containing solution passed; it reached 10% of the pre-loaded level (0.3 g MeBT/g GAC); this pattern did not change in response to increasing pH. MeBT liberation was estimated to be near 15–16% of the total MeBT loaded. However, MeBT in the effluent increased when challenged with trivalent Eu(III)—from 14 to 21% of its loading mass.

The influence of a pH increases on the desorption of CBT is summarized in Fig. 7b. CBT presence in the effluent was 10–15% of its pre-loaded level (0.19 g CBT/g GAC) for MRX-P and 25–30% of 0.17 g CBT/g GAC for MRX-Pox. For non-oxidized carbon, CBT was not detected in the effluent after 50 BV. Columns packed with oxidized GAC allowed 150 BV of Eu(III)-containing solution to pass at pH 4.5 before effluent CBT dropped below detection.

In summary, we executed both batch and flow through experiments; the batch experiments indicate capacity under static conditions, which more represent passive treatment scenarios. Alternatively, flow-through column experiments represent dynamic treatment scenario under conditions that can be limited by sorption kinetics. In a context of feasibility and validation, we make no formal comparisons between the respective treatment scenarios. Here, we simply use the time series of metal sequestration observed from batch exposures, to frame flow-through experiments. The expansion of hydrodynamic conditions to determine the thresholds for mass-transfer and kinetic limitations are the subject of optimization studies that are beyond the scope of this demonstrative work.

## Conclusions

It was demonstrated that acid oxidation of granular activated carbon (GAC), increased its oxygen content, lowered its pH_PZC_ value, improving its capacity for sorbing benzotriazole derivatives under acidic conditions. Oxidized GAC effectively retains benzotriazoles in a scenario which can be applied to treat metal radionuclides in acidic media in both batch and column systems, with a significant capacity for uranium, which reached a maximum for uranium elimination at 200 bed volumes (MRX-Pox) and 260 bed volumes (MRX-Pox + CBT). Redlich-Peterson models successfully described conditions under which MeBT and CBT were used to enhance GAC metal sequestration on activated carbon in acidic pH ranges. The results of this study suggest the importance of valence (charge) and ionic radius on the sorption potential. Results are consistent with a benzotriazole-metal complexation mechanism where benzotriazole(s) increase their affinity for GAC in a pH range where they approach charge neutrality, while at the same time coordinating aqueous heavy metals^35,﻿46﻿^. By taking into account the outcome of all breakthrough experiments, columns packed with CBT-impregnated oxidized activated carbons could be a cost-effective alternative for removing uranium and some of its daughter products from acidic environments.
